# Microbial Stimulation Reverses the Age-Related Decline in M Cells in Aged Mice

**DOI:** 10.1016/j.isci.2020.101147

**Published:** 2020-05-11

**Authors:** David S. Donaldson, Jolinda Pollock, Prerna Vohra, Mark P. Stevens, Neil A. Mabbott

**Affiliations:** 1 The Roslin Institute & Royal (Dick) School of Veterinary Sciences, University of Edinburgh, Easter Bush EH25 9RG, UK

**Keywords:** Microbiology, Microbiome

## Abstract

Aging has a profound effect on the immune system, termed immunosenescence, resulting in increased incidence and severity of infections and decreased efficacy of vaccinations. We previously showed that immunosurveillance in the intestine, achieved primarily through antigen sampling M cells in the follicle associated epithelium (FAE) of Peyer's patches, was compromised during aging due to a decline in M cell functional maturation. The intestinal microbiota also changes significantly with age, but whether this affects M cell maturation was not known. We show that housing of aged mice on used bedding from young mice, or treatment with bacterial flagellin, were each sufficient to enhance the functional maturation of M cells in Peyer's patches. An understanding of the mechanisms underlying the influence of the intestinal microbiota on M cells has the potential to lead to new methods to enhance the efficacy of oral vaccination in aged individuals.

## Highlights

• Exposure of aged mice to a young microbiota restores M cell density and function
• Systemic flagellin treatment enhances M cell density and function in aged mice
• Young microbiota exposure and flagellin treatment improve intestinal crypt function
• Microbial stimulation can restore the aging-related decline in M cells

## Graphical Abstract

**Figure:**
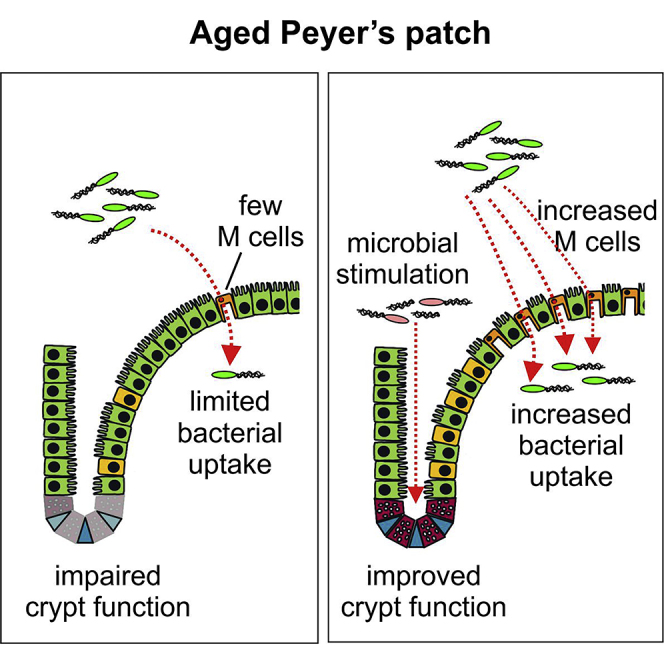

## Introduction

Maintaining the health of an increasingly aging population is an important challenge for society. The decline in immunity that occurs with aging, termed immunosenescence, results in decreased vaccine responses and an increased incidence and severity of pathogen infections. The intestinal microbiota is required for maturation of the immune system, but it can also have detrimental effects if it is dysbiotic. Although human intestinal microbiota is relatively stable for much of adulthood, aging induces significant shifts in its composition (Claesson et al., 2012). Therefore, restoring immunity in the intestines may have beneficial effects on both intestinal and systemic immune responses.

We have shown that M cell maturation in the intestine is dramatically reduced in aged mice (Kobayashi et al., 2013). M cells are specialized enterocytes normally present in the follicle associated epithelium (FAE) that overlies mucosal lymphoid tissues, such as the small intestinal Peyer's patches, and equivalents in the nasopharyngeal tract and lung (Date et al., 2017, Kimura et al., 2014, Kimura et al., 2019b, Mabbott et al., 2013). In the intestines, daughter cells derived from Lgr5+ intestinal stem cells at the intestinal crypt base differentiate into M cells upon receiving stimulation via the cytokine receptor activator of nuclear factor-κB ligand (RANKL) (de Lau et al., 2012) and expression of the transcription factors Spi-B (Kanaya et al., 2012) and Sox8 (Kimura et al., 2019a). M cells transport antigens from the mucosal surface into lymphoid tissues to immune cells that inhabit a unique pocket structure at their basal side (Kolesnikov et al., 2020, Komban et al., 2019). Mice lacking intestinal M cells have delayed development of mucosal antibody (IgA)-secreting plasma cell responses due to impaired Peyer's patch germinal center (GC) formation and T follicular helper (Tfh) cell differentiation (Rios et al., 2016) and develop more severe pathology when infected with intestinal pathogens such as *Citrobacter rodentium* (Nakamura et al., 2020). In the intestine, IgA production is thought to be a critical regulator of the intestinal microbiota and an important mediator against intestinal pathogens. Dysbiosis of the intestinal microbiota, as seen in humans (Catanzaro et al., 2019) and mice (Fagarasan et al., 2002) that lack IgA, is increasingly appreciated as an important factor affecting the development and/or progression of wide range of diseases (Carding et al., 2015). Thus, the delayed IgA response in M cell-deficient mice (Rios et al., 2016) is likely involved in maintaining a healthy microbiota and in immune responses against pathogens, both of which are reduced with aging.

Like humans, mice show age-related alterations in their microbiota that are thought to have negative consequences for health. The microbiota is not essential for M cell development, as germ-free mice have similar M cell densities to specific pathogen-free (SPF) mice (Kimura et al., 2015). However, other studies have shown that altering the microbiota may affect M cell development, suggesting that reduced M cell maturation in aged mice may be a consequence of age-related changes to the microbiota. For example, transferring SPF mice to conventional housing increased the M cell density in Peyer's patches (Smith et al., 1987). Short-term exposure of rabbit Peyer's patches to *Streptococcus pneumoniae* was also reported to have a similar effect (Borghesi et al., 1996). Impaired intestinal crypt function (Sehgal et al., 2018) or alterations to expression of RANKL, RANK (the receptor for RANKL), or the RANKL decoy receptor osteoprotegerin (OPG) (Kimura et al., 2020, Knoop et al., 2009) are each known to modify the density of M cells. However, it is unknown if these were altered in the above studies. Additionally, *Salmonella* Typhimurium may also alter the M cell density via the type III secretion system protein SopB (Tahoun et al., 2012) or by stimulating nociceptors on sensory neurons (Lai et al., 2020), strategies that may also be employed by members of the commensal microbiota to alter the M cell density.

Here, we tested if exposing aged mice to the microbiota from young mice would have an effect on M cell maturation in small intestinal Peyer's patches. We found that exposure to a young microbiota restored M cell maturation in aged mice and increased antigen uptake and IgA responses. Furthermore, the M cell density in aged mice could also be restored by stimulation with bacterial flagellin. Cells expressing olfactomedin 4 (OLFM4), a stem cell marker (van der Flier et al., 2009), in the intestinal crypts were increased in both conditions, suggesting that reduced M cell maturation in aged mice may be a consequence of an age-related decline in intestinal crypt function. By showing that the age-related decline in M cell maturation can be restored, it may be possible to reverse the age-related decline in mucosal vaccine efficacy and the ability to mount protective responses against intestinal pathogens.

## Results

### Passive Microbiota Transfer from Young Donors Enhances M Cell Development in Aged Mice

**Figure 1:**
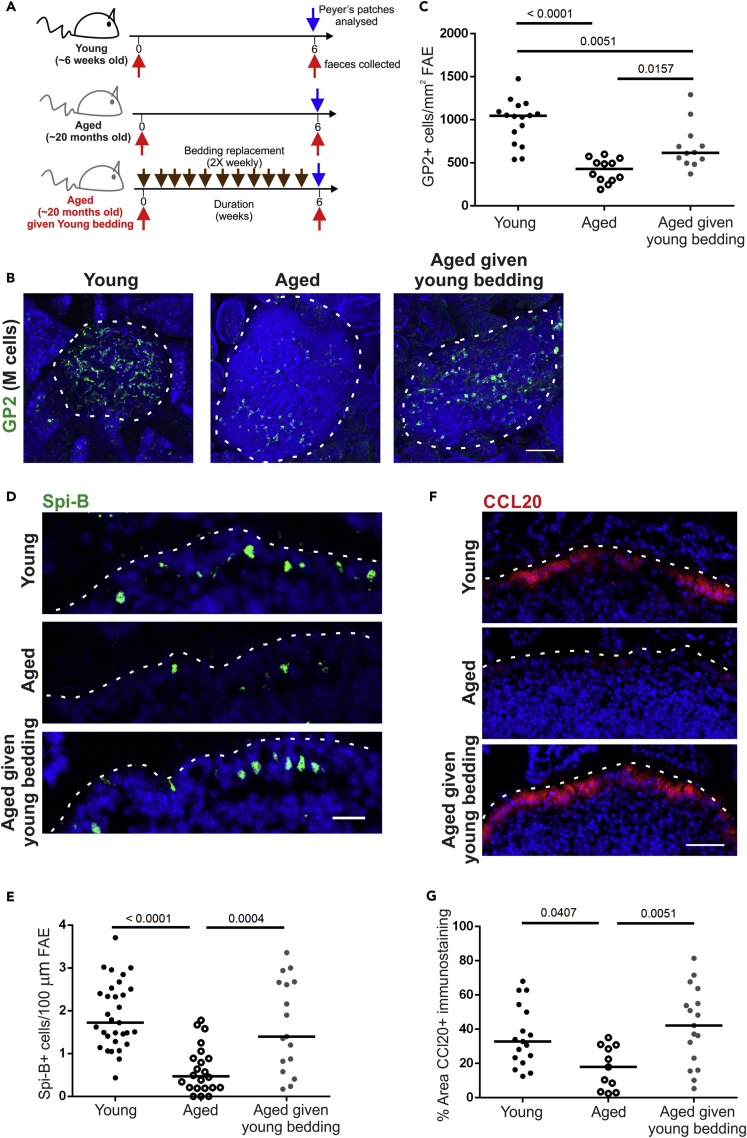
Passive Transfer of a Young Microbiota Enhances M Cell Development in Aged Mice (A) Cartoon describing the experimental setup. Aged mice (~20 months old) were housed for 6 weeks in cages of used bedding that had previously housed young mice. Bedding was replaced twice weekly. Control aged mice were housed in clean cages. (B) Whole-mount immunostaining of GP2+ M cells (green) in Peyer's patches from young, aged, and aged mice given young bedding. Counterstain, F-actin (blue). Scale bar, 100 μm. Broken line, the boundary of the FAE. (C) Quantitation of the number of GP2+ M cells in Peyer's patches from mice from each group. Each point represents an individual FAE. Horizontal line, median. n = 12–16/group from 3 to 4 mice. Statistical differences determined by one-way ANOVA. (D) Immunohistochemistry (IHC) detection of Spi-B+ cells (green) in the FAE of Peyer's patches from mice in each group. Nuclei detected using DAPI (blue). Scale bar, 20 μm. Broken line, the apical surface of the FAE. (E) Quantitation of the number of Spi-B+ cells in the FAE of Peyer's patches from mice from each group. Each point represents an individual section. Horizontal line, median. n = 17–31/group from 3 to 4 mice. Statistical differences determined by one-way ANOVA. (F) IHC detection of CCL20 (red) in the FAE of Peyer's patches from mice from each group. Nuclei detected using DAPI (blue). Scale bar, 20 μm. Broken line, the apical surface of the FAE. (G) Quantitation of the % area CCL20+ immunostaining in the FAE of Peyer's patches from mice from each group. Each point represents an individual FAE section. Horizontal line, median. n = 11–17/group from 3 to 4 mice. Statistical differences determined by one-way ANOVA.

The gut microbiota changes profoundly during aging and thus may have an indirect effect on M cell maturation. To explore this further, we facilitated the transfer of the microbiota from young mice into aged mice by housing them for 6 weeks in cages containing used bedding that had previously housed young mice. Groups of control aged mice were housed on clean bedding that had not previously been used to house other mice (Figure 1A). After 6 weeks, small intestines were excised from control aged mice, aged mice housed in young bedding, and young donor mice and the Peyer's patches whole-mount immunostained to detect glycoprotein 2 (GP2)+ cells, a marker of mature M cells (Hase et al., 2009, Kanaya et al., 2012).

As anticipated, the GP2+ M cell density was significantly reduced in aged mice compared with the young mice. However, in aged mice given young bedding, the GP2+ M cell density was significantly increased, albeit to a significantly lower level found in young mice (Figures 1B and 1C). M cell maturation is dependent on their intrinsic expression of the transcription factor Spi-B (Kanaya et al., 2012). In accordance with the increase in GP2+ M cells, the number of Spi-B+ cells in the FAE was also significantly higher in young mice and aged mice given young bedding (Figures 1D and 1E). This suggests that the decline in M cell maturity in aged mice may be partly dependent on age-related changes to the microbiota.

We previously showed that reduced M cell maturity in aged mice was associated with reduced CCL20 expression (Kobayashi et al., 2013), a chemokine produced by the FAE in response to RANKL stimulation. CCL20 is considered to contribute to M cell development by attracting chemokine receptor 6 (CCR6)-expressing lymphocytes to the FAE that stimulate M cell differentiation (Ebisawa et al., 2011). Immunostaining for CCL20 showed a significant reduction in CCL20 in aged mice compared with the young mice (Figures 1F and 1G), consistent with our previous data (Kobayashi et al., 2013). In contrast, aged mice given young bedding had a significantly higher level of CCL20 immunostaining in the FAE compared with control aged mice such that it was indistinguishable from the young mice (Figures 1F and 1G). Therefore, housing aged mice in used bedding from young mice restores CCL20 expression in the FAE.

### Microbiota Transfer from Young Donors Enhances Antigen Uptake and IgA Responses

**Figure 2:**
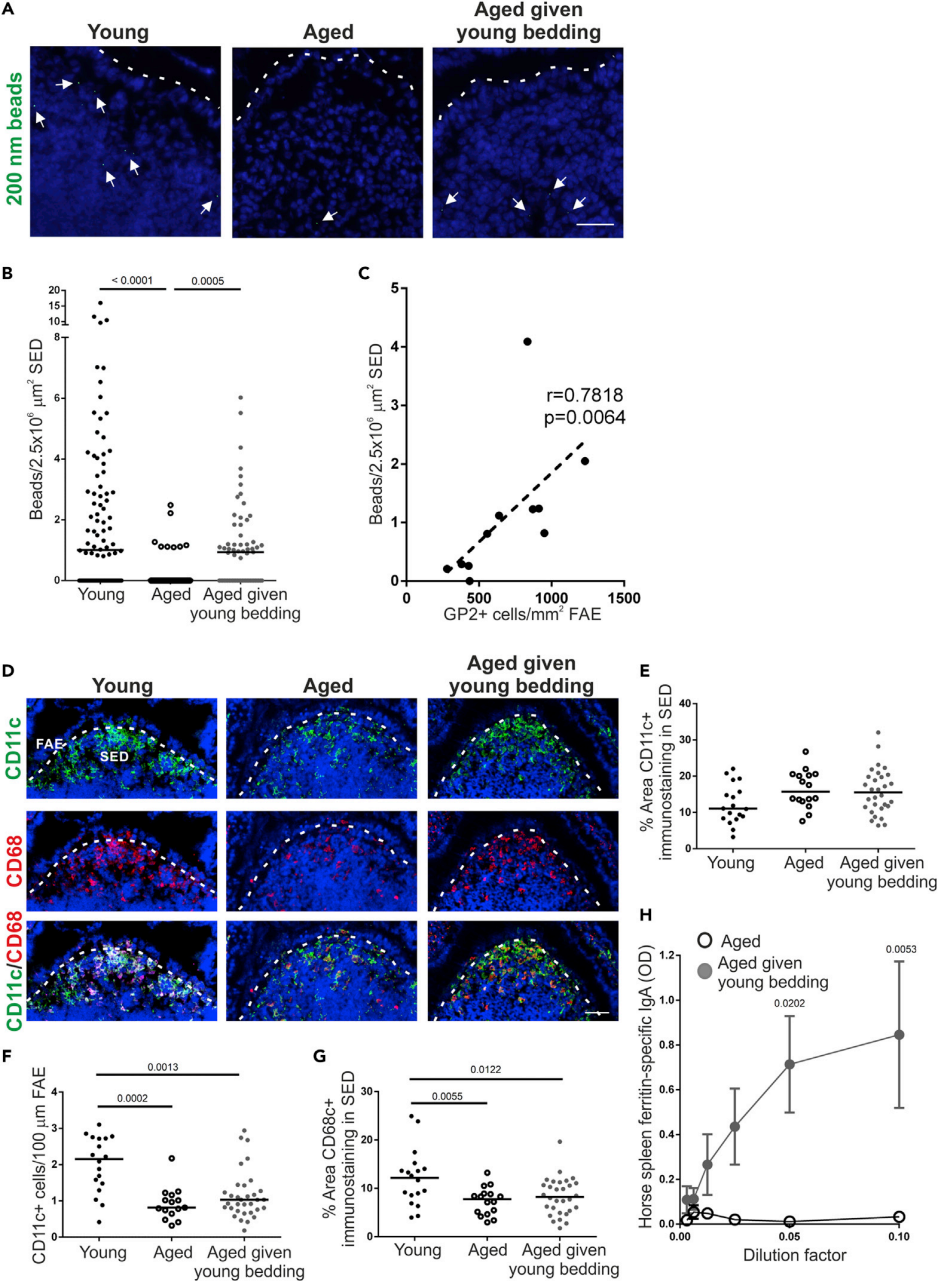
Passive Transfer of a Young Microbiota Enhances Uptake of Particulate Antigen into Aged Peyer's Patches (A) Histological detection of fluorescent 200-nm nanobeads (arrows) in the SED region of young, aged, and aged mice given young bedding. Scale bar, 100 μm. Broken line, the apical surface of the FAE. (B) Quantitation of the number of 200-nm fluorescent nanobeads in the SED of Peyer's patches from each group. Each point represents an individual section. Horizontal line, median. n = 51–94/group from 3 to 4 mice. Statistical differences determined by Kruskal-Wallis. (C) Correlation between GP2+ M cell-density in the FAE and the number of fluorescent 200-nm nanobeads in the SED. Statistical significance determined by Spearman correlation. (D) IHC detection on CD11c+ MNP (green) and CD68+ MNP (red) in the FAE and SED of Peyer's patches from mice from each group. Nuclei detected using DAPI (blue). Scale bar, 50 μm. Broken line, basal surface of the FAE. (E) The % area of CD11c+ immunostaining in the SED of mice from each group. Each point represents an individual section. Horizontal line, median. n = 16–30/group from 3 to 4 mice. Statistical differences determined by one-way ANOVA. (F) Quantitation of the number of CD11c+ MNP in the FAE of mice from each group. Each point represents an individual section. Horizontal line, median. n = 18–33/group from 3 to 4 mice. Statistical differences determined by Kruskal-Wallis. (G) Comparison of the % area of CD68+ immunostaining in the SED of mice from each group. Each point represents an individual section. Horizontal line, median. n = 16–30/group from 3 to 4 mice. Statistical differences determined by one-way ANOVA. (H) Specific anti-horse spleen ferritin (HSF) IgA levels were determined by ELISA 2 weeks after oral HSF administration in fecal homogenates from aged and aged mice given young bedding. Data presented as mean ± SEM. n = 3–4/group. Statistical differences determined by two-way ANOVA.

We next determined if the increased GP2+ M cell density in aged mice given young bedding also increased antigen uptake. Aged mice given young bedding were orally gavaged with 200-nm fluorescent nanobeads, a routinely used model for assessing M cell uptake ability (Knoop et al., 2009). The number of nanobeads transcytosed into the mononuclear phagocyte (MNP)-rich area directly beneath the FAE, known as the sub-epithelial dome (SED) region of the Peyer's patches, was then determined by microscopy. As anticipated (Kobayashi et al., 2013), significantly more nanobeads were found in the SED of young mice compared with aged mice (Figures 2A and 2B). However, consistent with the increased GP2+ M cell density, aged mice given young bedding had significantly more nanobeads in the SED than control aged mice (Figures 2A and 2B). Indeed, when these data were combined, a significant correlation between M cell density and mean nanobead uptake was observed (Figure 2C). The increased nanobead uptake was not associated with changes in MNP populations in the SED. Immunostaining for the MNP marker CD11c (integrin αX) revealed similar levels of CD11c + MNP in the SED of young mice, aged mice, and aged mice given young bedding (Figures 2D and 2E). Furthermore, both aged and aged mice given young bedding had reduced numbers of CD11c + MNP within the FAE (Figure 2F). Exposure to young bedding also did not restore the decreased levels of staining of another MNP marker, CD68, that was observed in aged mice (Figures 2D and 2G). These data suggest that alterations to the density of MNP populations in the SED were not responsible for the increased bead uptake in aged mice given young bedding.

In the same experiment, aged mice and aged mice given young bedding were administered horse spleen ferritin (HSF) as a model antigen via drinking water as described (Rios et al., 2016) to assess their ability to mount a mucosal antigen-specific IgA immune response. Specific anti-HSF IgA levels were determined by ELISA in fecal homogenates from each group prior to and 2 weeks after HSF administration. When corrected for non-specific IgA levels from samples taken prior to HSF administration, aged mice failed to mount a specific IgA response against this antigen. In contrast, aged mice given young bedding mounted a strong specific IgA response (Figure 2H). These data suggest that the increase in M cells in aged mice given young bedding results in increased antigen uptake and consequently an enhanced ability to mount an antigen-specific IgA response to an orally administered antigen.

### M Cell Density and Function Correlates with Increased Abundance of *Akkermansia muciniphila* and Decreased *Turicibacter* Species

**Figure 3:**
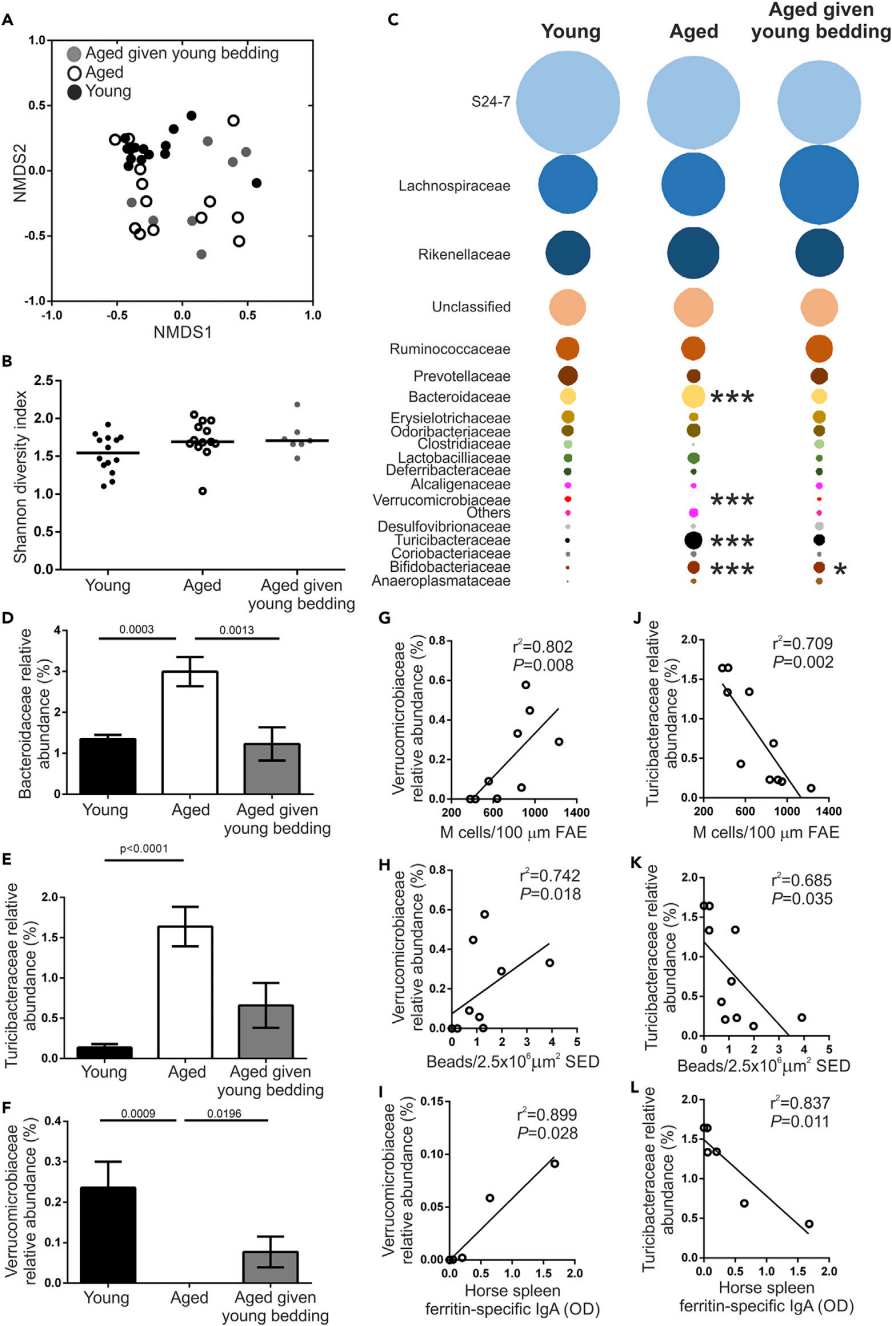
Changes to the Microbiota after Housing Aged Mice on Used Bedding from Young Mice (A) NMDS plot comparing fecal microbiota structure between mice from each age group. n = 7–14/group. Fecal microbiotas were significantly different between young mice and each group of aged mice (P = 0.002; HOMOVA). (B) Bacterial diversity in feces from young, aged, and aged mice given young bedding was compared using the Shannon index. Horizontal line, median. n = 7–14/group. Statistical differences determined by Kruskal-Wallis. (C) Bubble plot showing the relative abundance of distinct bacterial families in the fecal microbiotas of mice from each group. n = 7–14/group. Statistical differences for each family determined by one-way ANOVA or Kruskal-Wallis where appropriate. ∗ P < 0.05; ∗∗∗ P < 0.001. (D–F) The relative abundance of (D) *Bifidobacteriaceae*, (E) *Turicibacteriaceae*, and (F) *Verrucomicrobiaceae* in feces of young, aged, and aged mice given young bedding. Data presented as mean ± SEM. n = 7–14/group. Statistical differences determined by Kruskal-Wallis. (G–I) Correlation between the relative abundance of *Verrucomicrobiaceae* in feces and (G) the density of FAE GP2+ M cells, (H) the number of fluorescent 200-nm nanobeads in the SED, and (I) the level of antigen-specific fecal IgA. Statistical significance determined by Spearman correlation. (J–L) Correlation between the relative abundance of *Turicibacteriaceae* in the feces and (J) the density of FAE GP2+ M cells, (K) the number of fluorescent 200-nm nanobeads detected in the SED, and (L) the level of antigen-specific fecal IgA. Statistical significance determined by Spearman correlation.

To determine the changes to the intestinal microbiota that occurred following exposure of aged mice to used bedding from young mice, DNA was extracted from fecal pellets from aged mice, aged mice given young bedding, and the young donor mice to assess microbiota alpha diversity, structure, and relative bacterial abundances using 16S rRNA gene metabarcoding as described (Pollock et al., 2018). The advantage of using relative abundance calculations is that the number of sequences can be normalized across samples, to allow robust descriptive and statistical analyses to be carried out. Analysis of molecular variance (AMOVA) testing revealed a significant difference in microbiota structure between young and aged mice (P = 0.001). However, no significant difference was observed between aged mice and aged mice given young bedding (P = 0.07). Differences in microbiota structure were visualized using non-metric multidimensional scaling (NMDS; Figure 3A). The microbiotas from the young mice formed a tight cluster, whereas both the aged mice and the aged mice given young bedding did not. This suggested greater variation between individuals in the microbiota composition with increased age, confirmed using homogeneity of variance (HOMOVA) testing (P = 0.002). Interestingly, no difference in Shannon diversity was observed between the young and aged mice (Figure 3B). This indicates that the exposure of aged mice to used bedding from young mice did not result in a large change in microbiota alpha diversity. These data are consistent with data from a similar study of aged C57BL/6 mice exposed to young bedding that also reported that aging was not associated with a reduction in bacterial diversity (Stebegg et al., 2019).

The microbiota was then analyzed at the family level to look for differences between experimental groups (Figure 3C). A significant increase in the relative abundance of *Bifidobacteriaceae* in both groups of aged mice was observed (Figure 3D). The relative abundances of both *Bacteriodaceae* and *Turicibacteriaceae* were significantly increased with aging and were reduced in the aged mice given young bedding (Figures 3D and 3E). Conversely, the relative abundance of *Verrucomicrobiaceae* was decreased with aging and significantly increased in aged mice given young bedding (Figure 3F). Of these three bacterial families, only the *Verrucomicrobiaceae* and *Turicibacteriaceae* correlated consistently with the functional changes observed in aged mice given young bedding. The relative abundance of *Verrucomicrobiaceae*, represented by the single species *Akkermansia muciniphila*, demonstrated a significant positive correlation with M cell density, nanobead uptake and fecal anti-HSF IgA responses (Figures 3G–3I). Likewise, the relative abundance of *Turicibacteriaceae*, representing a single unclassified *Turicibacter* species, negatively correlated with these parameters (Figures 3J–3L). Therefore, an increased abundance of *A*. *muciniphila* and a decreased abundance of *Turicibacter* species correlate with the decline in M cell maturation observed in aged mice.

### Systemic Flagellin Treatment Enhances M Cell Development in Aged Mice

We previously showed that the ageing-related decline in M cell maturation was associated with reduced CCL20 expression in the FAE (Kobayashi et al., 2013). Mice deficient in CCR6, the receptor for CCL20, also show reduced M cell maturation (Kobayashi et al., 2013) highlighting the importance of this chemokine in M cell development. The restored functional capacity of M cells in aged mice given young bedding was associated with a restoration of CCL20 expression in the FAE equivalent to that of young mice. Therefore, strategies that boost CCL20 expression in the FAE may directly reverse the decline in M cell maturation in aged mice. Systemic administration of flagellin has been shown to enhance CCL20 expression in the FAE and villous epithelium (Sirard et al., 2009). Furthermore, co-administration of flagellin and nanobeads into the lumen of ligated Peyer's patches enhanced particle uptake, suggesting a direct modulation of M cell activity (Chabot et al., 2008). Therefore, we hypothesized that systemic flagellin administration may enhance M cell maturation in aged mice.

**Figure 4:**
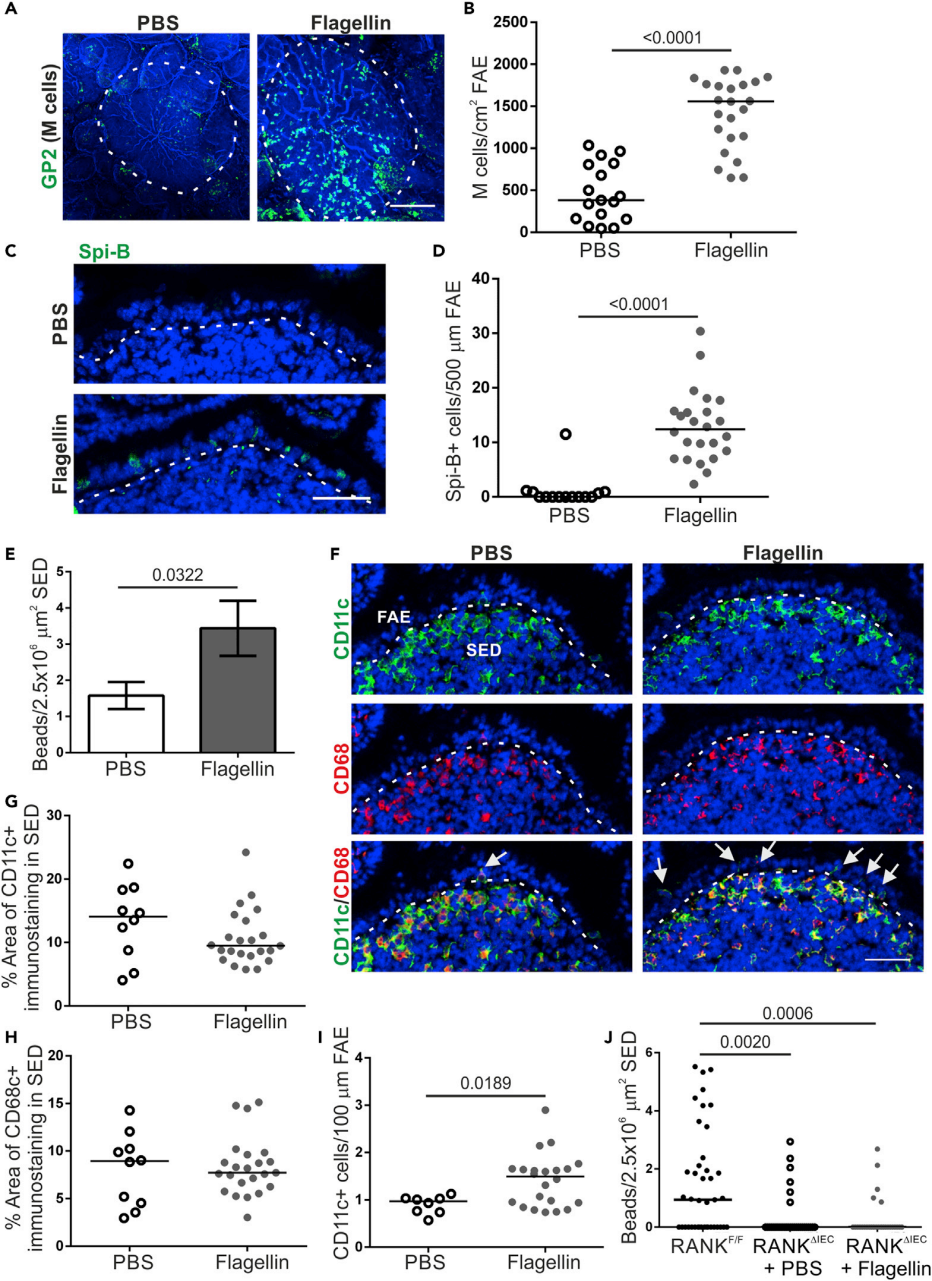
Systemic Flagellin Treatment Enhances M Cell Development in Aged Mice (A) Whole-mount immunostaining of GP2+ M cells (green) in Peyer's patches from PBS- and flagellin-treated aged mice. Counterstain, F-actin (blue). Scale bar, 100 μm. Broken line, the boundary of the FAE. (B) Quantitation of the number of GP2+ M cells in Peyer's patches from mice from each group. Each point represents an individual FAE. Horizontal line, median. n = 17–23/group from 3 to 4 mice. Statistical difference determined by t test. (C) IHC detection on Spi-B+ cells (green) in the FAE of Peyer's patches from mice from each group. Nuclei detected with DAPI (blue). Scale bar, 20 μm. Broken line, the apical surface of the FAE. (D) Quantitation of Spi-B+ cells in the FAE of Peyer's patches from mice in each group. Each point represents an individual section. Horizontal line, median. n = 14–24/group from 3 to 4 mice. Statistical difference determined by Mann-Whitney. (E) Quantitation of the number of 200-nm fluorescent nanobeads in the SED of PBS- and flagellin-treated aged mice 24 h after oral exposure. Data presented as mean ± SEM. n = 53–90/group from 3 to 4 mice. Statistical difference determined by Mann-Whitney. (F) IHC detection on CD11c+ (green) and CD68+ MNP (red) in the FAE and SED in mice from each group. Nuclei detected with DAPI (blue). Scale bar, 50 μm. Broken line, the basal surface of the FAE. Arrows, CD11c+ cells in FAE. (G and H) The % area of CD11c+ (G) and CD68+ (H) immunostaining in the SED of mice in each group. Each point is from an individual section. Horizontal line, median. n = 10–23/group from 3 to 4 mice. Statistical difference determined by Mann-Whitney. (I) Number of CD11c+ cells in the FAE of Peyer's patches from PBS- and flagellin-treated aged mice. Each point is from an individual section. Horizontal line, median. n = 8–23/group from 3 to 4 mice. Statistical difference determined by t test. (J) Quantitation of the number of 200-nm fluorescent nanobeads in the SED of PBS or flagellin-treated M cell-deficient RANK^ΔIEC^ mice 24 h after oral exposure. M cell-sufficient RANK^F/F^ mice were used as a positive control. Each point is from an individual section. Horizontal line, median. n = 31–41/group from 3 mice. Statistical differences determined by Kruskal-Wallis.

Aged mice were injected intraperitoneally (IP) with 50 ng of flagellin for 3 days, consistent with similar protocols for enhancing M cell development via systemic RANKL administration (Donaldson et al., 2016, Kanaya et al., 2012, Knoop et al., 2009). Control aged mice were injected with PBS. After 3 days, Peyer's patches were excised and whole-mount immunostained for GP2. Flagellin administration induced a significant increase in the GP2+ M cell density in aged mice that exceeded the density routinely observed in young C57BL/6J mice in our facility (Figures 4A and 4B). A significant increase in Spi-B+ cells was also observed in the FAE after flagellin treatment (Figures 4C and 4D). Therefore, systemic flagellin administration is sufficient to reverse the decline in GP2+ M cells observed in the FAE of Peyer's patches from aged mice.

### Systemic Flagellin Treatment Enhances M Cell-Mediated Antigen Uptake in Aged Mice

To confirm that increased M cells in aged mice following flagellin treatment enhanced the uptake of luminal antigens, aged mice were treated with flagellin as above and orally gavaged with fluorescent nanobeads on day 3 of flagellin treatment. Nanobead uptake into the SED of Peyer's patches was compared 24 h later. Consistent with the increase in M cell density, a significant increase in the number of nanobeads in the SED was observed in aged mice treated with flagellin compared with PBS-treated controls (Figure 4E). The increased nanobead uptake observed in flagellin-treated aged mice was not due to altered MNP populations within the SED as analysis of the MNP markers CD11c and CD68 by immunostaining did not show any difference between PBS and flagellin-treated mice (Figures 4F–4H). However, the number of CD11c+ cells within the FAE was significantly increased after flagellin treatment, suggesting enhanced interactions between CD11c+ cells and M cells (Figure 4I).

To exclude the possibility that the flagellin-mediated increase in particle uptake was due to M cell-independent uptake mechanisms such as the increased abundance of CD11c+ cells in the FAE, we took advantage of RANK^ΔIEC^ mice, which have a specific deficiency in RANK expression in the intestinal epithelium and lack M cells (Rios et al., 2016). RANK^ΔIEC^ mice were treated as above with flagellin (or PBS as a control) and orally gavaged with fluorescent nanobeads. M cell-sufficient RANK^FL/FL^ mice were also orally gavaged with nanobeads as a positive control. Consistent with previous studies (Donaldson et al., 2016, Rios et al., 2016), significantly fewer nanobeads were detected in the SED of Peyer's patches from flagellin-treated RANK^ΔIEC^ mice compared with RANK^FL/FL^ mice (Figure 4J). Since flagellin treatment did not enhance the uptake of nanobeads in RANK^ΔIEC^ mice, this suggests that the effect of flagellin on antigen uptake into aged Peyer's patches was due to the flagellin-mediated increase in M cell density.

**Figure 5:**
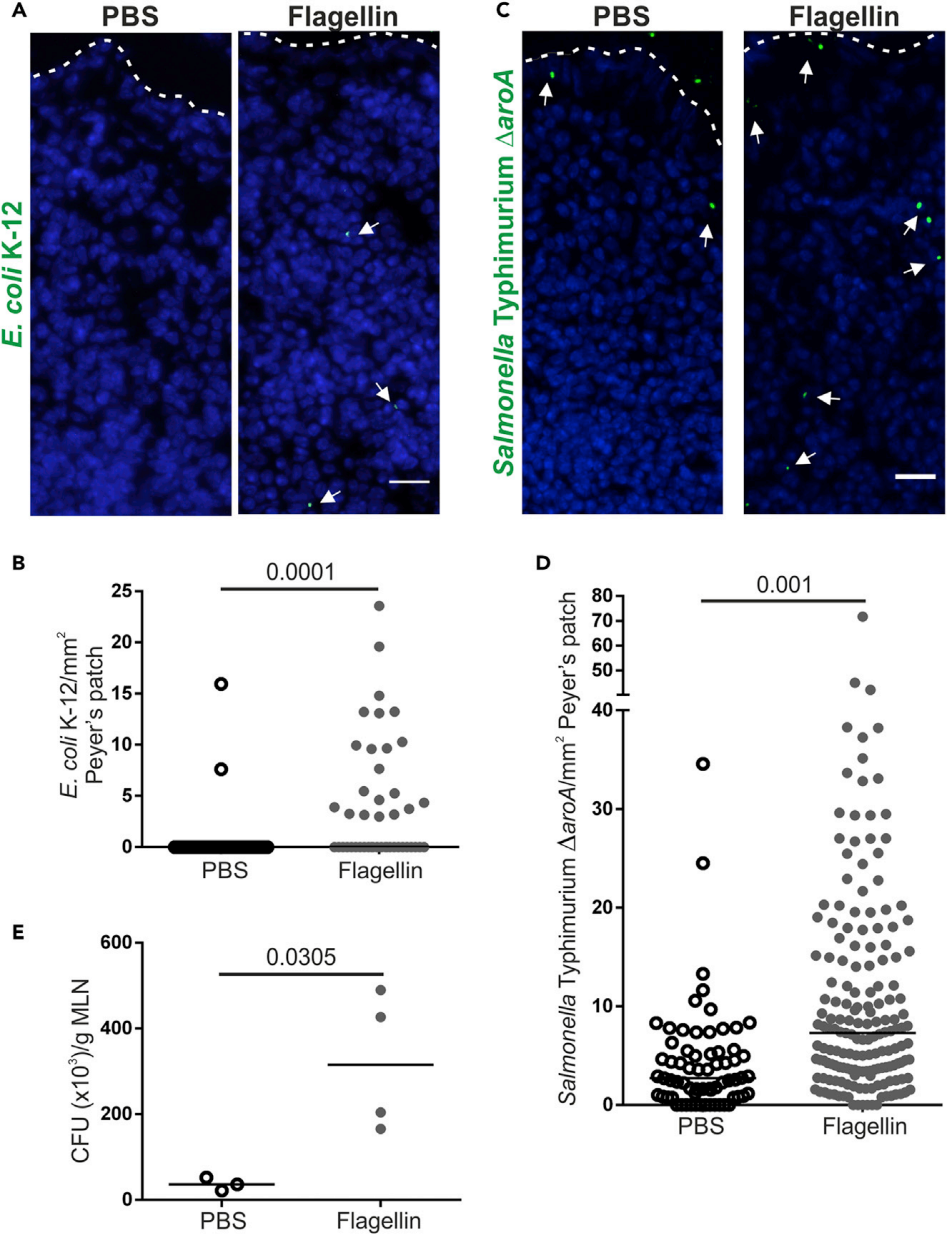
Systemic Flagellin Treatment Enhances Uptake of FimH + Bacteria into Aged Peyer's Patches (A–E) Aged mice (n = 3–4) were treated with flagellin (or PBS, control) and GFP-expressing *E*. *coli* K12 or *Salmonella* Typhimurium injected into ligated loops containing a Peyer's patch; 1.5 h later the abundance of these bacteria in the SED of Peyer's patches and MLN was quantified. (A) IHC detection of GFP-expressing *E*. *coli* K-12 (arrows) in the SED. Nuclei detected using DAPI (blue). Broken line, apical FAE surface. Scale bar, 50 μm. (B) Quantitation of the number of GFP-expressing *E*. *coli* K-12 in the SED of PBS- and flagellin-treated mice. Each point is from an individual section. Horizontal line, median. n = 36–45/group from 3 to 4 mice. Statistical difference determined by Mann-Whitney. (C) IHC detection of GFP-expressing *S*. Typhimurium Δ*aroA* (arrows) in the SED. Nuclei detected using DAPI (blue). Broken line, apical FAE surface. Scale bar, 50 μm. (D) Quantitation of the number of GFP-expressing *S*. Typhimurium Δ*aroA* in the SED of PBS- and flagellin-treated mice. Each point is from an individual section. Horizontal line, median. n = 67–171/group from 3 to 4 mice. Statistical difference determined by Mann-Whitney. (E) Quantitation of *S*. Typhimurium Δ*aroA* colony-forming units (CFU)/g in MLN of PBS- and flagellin-treated mice. Points are MLN from individual mice. Horizontal line, median. n = 3–4/group. Statistical difference determined by t test.

M cells express a range of receptors on their apical surface that mediate the specific update of distinct pathogenic microorganisms or their toxins (Mabbott et al., 2013). For example, GP2 enables M cells to acquire certain FimH-expressing pathogenic bacteria (Hase et al., 2009). To determine if flagellin treatment also increased M cell-mediated uptake of bacteria, aged mice were treated as above with flagellin (or PBS as a control) and GFP-expressing non-invasive *Escherichia coli* K-12 or invasive *Salmonella* Typhimurium Δ*aroA* (both of which are known to be taken up by M cells via FimH-GP2 interactions) were injected into the lumen of ligated Peyer's patches. Consistent with the increased uptake of nanobeads, flagellin induced a significant increase in the uptake of *E*. *coli* K-12 (Figures 5A and 5B) and *S*. Typhimurium Δ*aroA* (Figures 5C and 5D) into the SED of the Peyer's patches. Dissemination of these bacteria to the mesenteric lymph nodes (MLN) was also determined. As anticipated, the non-invasive *E*. *coli* K-12 was not detectable in the MLN. However, consistent with the significant increase in *S*. Typhimurium Δ*aroA* uptake in Peyer's patches of flagellin-treated aged mice, a significantly higher abundance of *S*. Typhimurium Δ*aroA* was recovered from the MLN of the flagellin-treated aged mice (Figure 5E). Thus, flagellin treatment enhances the uptake of FimH + bacteria into Peyer's patches.

### Flagellin Does Not Enhance M Cell Development in Intestinal Organoids *In Vitro*

**Figure 6:**
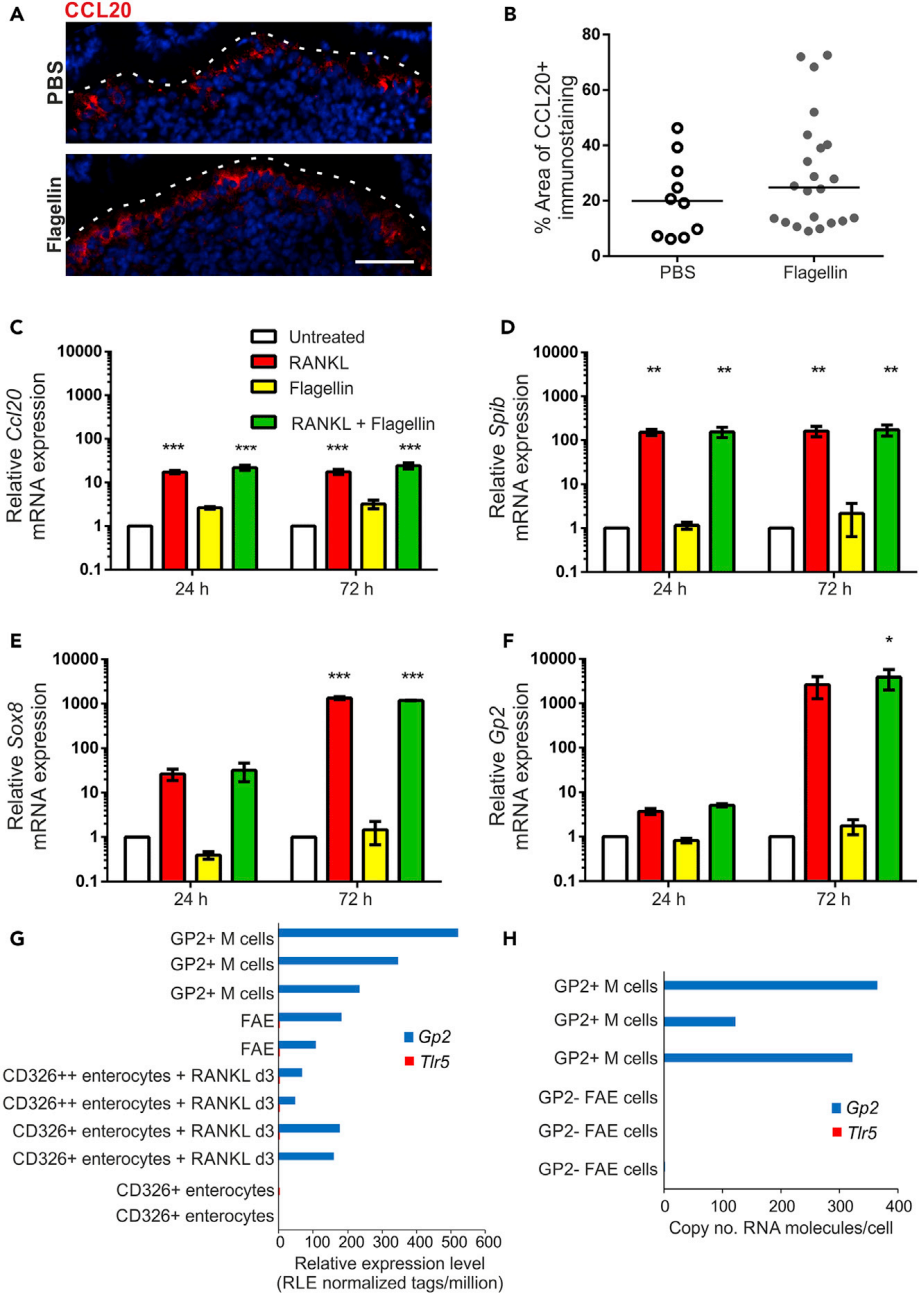
Flagellin Treatment Does Not Enhance M Cell Differentiation in Enteroids (A) IHC detection of CCL20 (red) in the FAE of Peyer's patches from PBS- and flagellin-treated aged mice. Nuclei detected with DAPI (blue). Scale bar, 20 μm. Broken line shows the apical FAE surface. (B) Quantitation of the % area CCL20+ immunostaining in the FAE of Peyer's patches from mice in each group of (A). Each point is from an individual FAE. Horizontal line, median. n = 10–22/group from 3 to 4 mice. Statistical difference determined by Mann-Whitney. (C–F) *In vitro* enteroids prepared from small intestinal crypts were treated with RANKL, flagellin or both. Expression of *Ccl20* (C), *Spib* (D), *Sox8* (E), and *Gp2* (F) was compared by RT-qPCR 24 or 72 h later. Mean expression levels were normalized so that untreated enteroids at 24 h equaled 1.0. Data expressed as mean ± SEM. n = 3. Statistical differences determined by two-way ANOVA. (G and H) Comparison of *Tlr5* and *Gp2* mRNA expression (G) in individual cell populations in deep CAGE sequence data from the FANTOM5 project of the FANTOM consortium (Forrest, 2014), and (H) in published mRNA-seq data from isolated GP2+ M cells (NCBI Gene Expression Omnibus: GSE108529 [Kimura et al., 2020]).

Although an independent study reported that systemic flagellin-treatment increased CCL20 expression in the FAE (Sirard et al., 2009), our analysis showed no difference in the FAE of aged mice after flagellin treatment (Figures 6A and 6B). We explored this further using *in vitro* enteroids prepared from small intestinal crypts. RANKL-treatment induced high expression of *Ccl20* mRNA in enteroids (mean 17.4-fold), whereas flagellin-treatment induced only low levels of expression (mean<3-fold) that was not significantly different from untreated enteroids (Figure 6C). Furthermore, treatment of enteroids with RANKL and flagellin did not enhance *Ccl20* expression above RANKL-treatment alone (Figure 6C). Flagellin was also unable to induce expression of the M cell-related genes *Spib*, *Sox8*, or *Gp2* in enteroids and did not enhance the RANKL-mediated increase in expression of these genes (Figures 6D–6F). Flagellin stimulates host cells through binding to Toll-like receptor 5 (TLR5). The lack of significant induction of *Ccl20* and M cell-related gene expression in enteroids after flagellin treatment was further supported by the absence of *Tlr5* mRNA expression in deep CAGE sequence data from GP2+ M cells, FAE and RANKL-stimulated enterocytes from the FANTOM consortium (Figure 6G [Forrest, 2014]), and in published mRNA sequencing (mRNA-seq) data from isolated GP2+ M cells (Figure 6H [Kimura et al., 2020]). These data suggest that the effect of flagellin on M cell density *in vivo* is not mediated through direct TLR5-mediated stimulation of enterocytes or immature M cells in the FAE.

### Exposure of Aged Mice to a Young Microbiota or Systemic Flagellin Treatment Restores Small Intestinal Crypts

M cells develop from Lgr5+ intestinal stem cells at the intestinal crypt base (de Lau et al., 2012), and these stem cells are supported by Paneth cells (Sato et al., 2011). Age-related defects in the regenerative ability of intestinal epithelium are thought to arise from impaired Paneth cell function that reduces their ability to support Lgr5+ stem cells (Pentinmikko et al., 2019). Interestingly, studies using transgenic reporter mice show that, in the small intestinal epithelium, TLR5 expression is restricted to Paneth cells, consistent with above data showing no TLR5 expression in the FAE or M cells. This suggested that the restoration of M cell maturation in aged mice by flagellin may be Paneth cell dependent.

**Figure 7:**
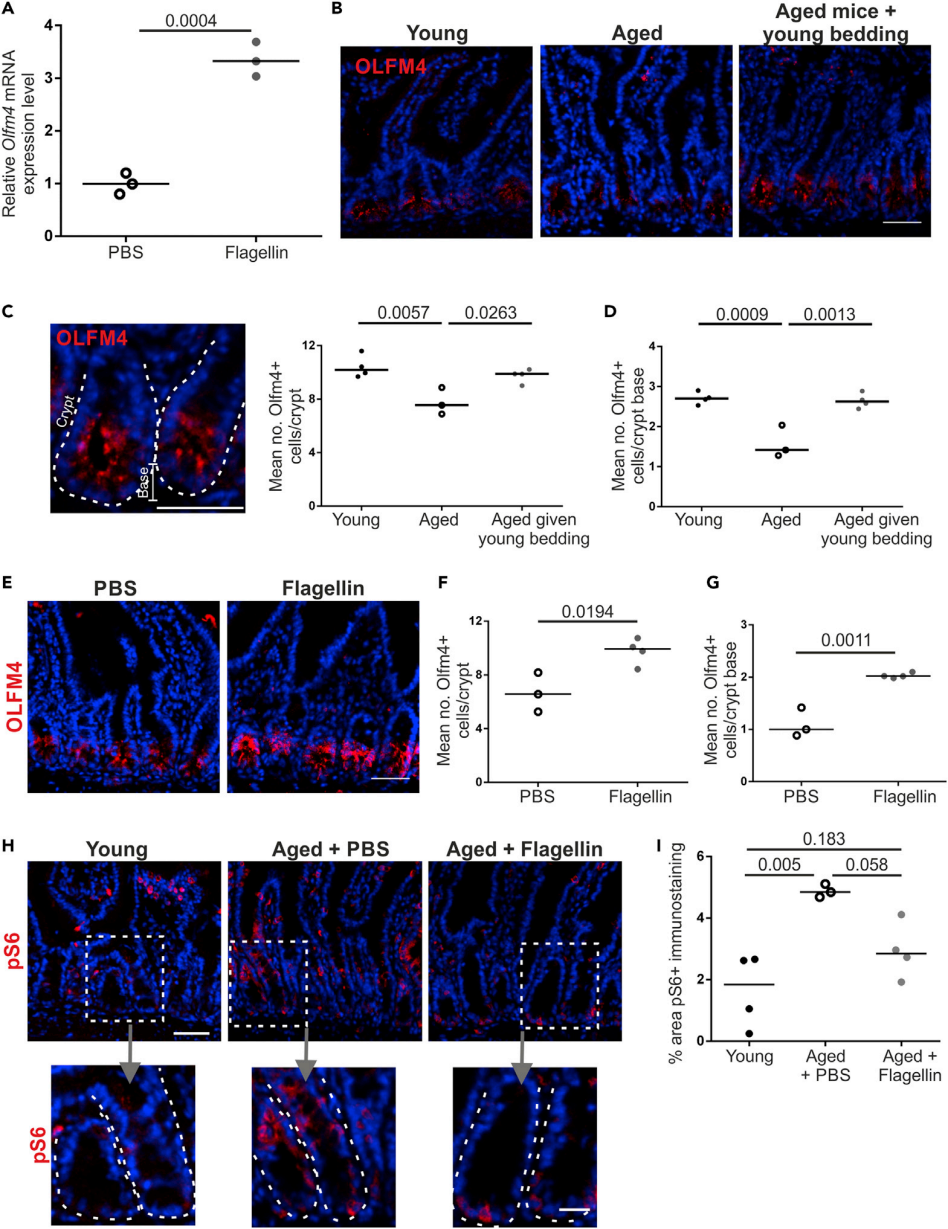
Exposure of Aged Mice to a Young Microbiota or Flagellin Restores Small Intestinal Crypts (A) Olfm4 mRNA expression was compared by RT-qPCR 24 h after flagellin stimulation of *in vitro* enteroids prepared from small intestinal crypts. Horizontal line, median. n = 3/group. Statistical difference determined by t test. (B) IHC detection of OLFM4 (red) in small intestinal crypts of young, aged, and aged mice given young bedding. Nuclei detected with DAPI (blue). Scale bar, 50 μm. (C and D) Quantitation of the number of OLFM4+ cells in small intestinal crypts (C) and the crypt base (D) from mice in each group in (B) Each point is the mean of an individual mouse. n = 3–4/group. Horizontal line, median. Statistical differences determined by one-way ANOVA. (E) IHC detection of OLFM4 in small intestinal crypts from PBS- and flagellin-treated aged mice. Nuclei detected with DAPI (blue). Scale bar, 50 μm. (F and G) Quantitation of the number of OLFM4+ cells in small intestinal crypts (F) and the crypt base (G) from mice in each group in (E). Each point is the mean of an individual mouse. n = 3–4/group. Horizontal line, median. Statistical differences determined by t test. (H) IHC detection of pS6 (red) in small intestinal crypts from young, PBS-treated aged, and flagellin-treated aged mice. Nuclei detected with DAPI (blue). Scale bar: upper panels, 50 μm; lower panels, 20 μm. (I) Quantitation of the % area pS6+ immunostaining in small intestinal crypts of mice in each group in (H). Each point is the mean of an individual mouse. n = 3–4/group. Horizontal line, median. Statistical differences determined by t test.

Stimulation of enteroids with flagellin has been shown to increase the expression of OLFM4 (Price et al., 2018), a marker of intestinal crypt stem cells (van der Flier et al., 2009). Stimulation of our enteroids with flagellin confirmed a significant increase in *Olfm4* mRNA expression (Figure 7A). To determine if this occurred *in vivo*, the number of OLFM4+ cells/crypt was determined in sections of small intestine from young, aged, and aged mice given young bedding (Figure 7B). OLFM4+ cells were observed in both the transamplifying region and the base of the crypt where the Lgr5+ stem cells reside. As anticipated, the total number of OLFM4+ cells/crypt and number of OLFM4+ cells at the base of the crypt of aged control mice was significantly lower than in young mice (Figures 7C and 7D). However, the number of OLFM4+ cells/crypt and at the crypt base was significantly increased in aged mice given young bedding to levels equivalent to young mice. Likewise, a significant increase in OLFM4+ cells/crypt and at the crypt base was observed in flagellin-treated aged mice compared with PBS-treated controls (Figures 7E–7G). Paneth cell dysfunction in aging has been linked to increased mTORC1 (mammalian target of rapamycin complex 1) activity in mice (Pentinmikko et al., 2019). Phosphorylated ribosomal protein S6 (pS6) is a downstream effector of mTORC1 that is increased in aged Paneth cells (Figures 7H and 7I) (Pentinmikko et al., 2019). The crypts of PBS-treated aged mice had a significantly higher area that stained positive for pS6 compared with young mice (Figures 7H and 7I). Consistent with the increased number of OLFM4+ cells, the area that stained positive for pS6 in the crypts of flagellin-treated aged mice was not significantly to that observed in young mice. Together, these data suggest that the increased M cell maturation in aged mice induced by both exposure to young bedding and flagellin may have been due to restored function of the intestinal crypts.

## Discussion

The decline in immunity with age significantly affects the health and well-being of the elderly. Here, we show that the age-related decline in M cell maturation can be restored by flagellin stimulation or exposure to the microbiota of young mice. The restoration of M cell maturation in aged mice resulted in enhanced antigen uptake and intestinal IgA responses against a model antigen. M cells develop from stem cells located in the intestinal crypts (de Lau et al., 2012). Interestingly, both exposure to a young microbiota and flagellin stimulation increased the numbers of OFLM4+ cells in the intestinal crypts, suggesting that improving crypt function may be important in re-establishing intestinal immunity in aging.

Although the M cell density was increased in aged mice given young bedding, factors other than the microbiota may have played a role. Housing male mice in used bedding of other male mice is known to alter behavior, including increased aggression, via pheromones found in urine (Lacey et al., 2007). However, olfactory sensing also declines with age (Patel and Larson, 2009) and no increased aggression was noted in the aged mice given young bedding in our study, suggesting they may have been unable to detect the pheromones. Additionally, no direct effect of pheromones or urine on immunity has been demonstrated. Frequent cage changing can induce stress (Rasmussenm et al., 2011); however, the aged mice given clean bedding were given equivalent cage changes. Housing aged mice in used bedding from germ-free mice would help to resolve the potential contribution of these factors.

Mice lacking M cells have impaired fecal IgA responses (Rios et al., 2016). The age-related decline in M cell maturation is likely to affect the development of IgA responses against the microbiota. IgA plays an important role in regulating the gut microbiota (Catanzaro et al., 2019, Fagarasan et al., 2002), thus the decline in M cell maturation could contribute to the age-related changes in the microbiota. Reversing the decline in M cell maturation may beneficially alter the aged microbiota composition and reduce the age-associated inflammation which the aged microbiota is known to induce (Fransen et al., 2017). This could also have a beneficial effect on the increasing number of diseases whose development and/or progression are influenced by the intestinal microbiota. An important factor in this may be how long the increase in M cell maturation lasts. Flagellin stimulation likely induces a transient increase in M cell maturation, although it is possible that this may be enhanced by subsequent treatments. Whether the increase in M cell maturation is sustained in aged mice given young bedding following their transfer back to clean bedding is unknown.

Mice lacking M cells develop more severe pathology when infected with *C*. *rodentium* (Nakamura et al., 2020), paralleling the increased severity of intestinal infections observed with age. This suggests that increasing M cell maturation in aging may reduce the severity of these infections. Additionally, M cells are an attractive target as a means to induce IgA in vaccinations (Yamamoto et al., 2011). Increasing M cell maturation in aged individuals, even transiently via flagellin treatment, would likely increase the efficacy of M cell targeted vaccines. Flagellin would also have the added benefit of having adjuvant properties that would boost the immune response (Hajam et al., 2017).

IgA responses depend on the development of GC responses in the Peyer's patch, and GC B and Tfh cells are also reduced in M cell-deficient mice (Rios et al., 2016). Consistent with the decline in M cell maturation (Kobayashi et al., 2013), Peyer's patch GC B and Tfh cells are also reduced in aged mice (Stebegg et al., 2019). The decline in Peyer's patch GC B and Tfh cells in aged mice could be reversed by transfer of a young microbiota (Stebegg et al., 2019), consistent with the results of this study. It is therefore likely that the increased GC B and Tfh cells observed in aged mice after young microbiota transfer is dependent on increased M cell maturation.

M cell maturation results in the ability to transcytose antigens. Increased antigen trafficking into the Peyer's patch would stimulate naive lymphocytes. This could be enhanced by activated antigen-specific B cells that can sample antigens directly from M cells and migrate to the GC (Komban et al., 2019). Additional mechanisms beyond antigen transcytosis could also contribute to increased IgA production and GC reactions in aged mice. M cells also sample nanoparticles that capture microbiota-derived immune-stimulatory macromolecules such as peptidoglycan (Powell et al., 2015). Interestingly, this drives expression of programmed death-ligand 1 (PD-L1) in the Peyer's patch, which may signal to Tfh cells through the receptor PD-1, an absence of which alters the GC response and results in poor IgA selection (Kawamoto et al., 2012). Additionally, other bacterial ligands may be important in stimulating Tfh development and IgA responses. For example, germ-free mice containing T cells that lack TLR signaling have reduced Tfh cell development in response to a TLR2 agonist and fail to mount specific intestinal IgA responses (Kubinak et al., 2015). Therefore, bacterial ligands transported into Peyer's patches of aged mice alongside antigens could effectively act as adjuvants that boost the GC reaction and specific IgA development.

Our analysis of changes to the fecal microbiota of aged mice given young bedding revealed a positive correlation between M cells, bead uptake, and the specific IgA response with the abundance of *A*. *muciniphila* and a negative correlation between the same parameters and an unclassified *Turicibacter* species. Whether changes in the abundance in these species contributes to the increase in M cell maturation or are merely representative of broader changes to the microbiota associated with the restoration of immunity is unknown. The abundance of *A*. *muciniphila* is also decreased in aged humans (Collado et al., 2007), and reduced *A*. *muciniphila* is associated with the development of inflammation in germ-free mice reconstituted with an aged microbiota (Fransen et al., 2017). *A*. *muciniphila* can enhance epithelial integrity *in vitro* (Reunanen et al., 2015) and upregulate genes involved in metabolism in enteroids (Lukovac et al., 2014), suggesting the increased abundance in aged mice given young bedding may have a direct effect on epithelial cells and M cell maturation. In support of this, a recent study showed that nicotinamide produced by *A*. *muciniphila* was protective in a model of amyotrophic lateral sclerosis (Blacher et al., 2019). Nicotinamide is a subunit of nicotinamide adenine dinucleotide (NAD^+^). Treatment of aged mouse enteroids with a NAD+ precursor, nicotinamide riboside, restores the impaired growth of aged enteroids (Igarashi et al., 2019). Interestingly, nicotinamide riboside also restores the reduction in OLFM4+ cells in the crypts of aged mice *in vivo* (Igarashi et al., 2019) as seen in our aged mice given young bedding, suggesting a potential link between *A*. *muciniphila*-produced metabolites and enhanced M cell maturation in aged mice.

However, the correlation between *A*. *muciniphila* and M cells may have been due to a common underlying mechanism rather than a direct link between the two. *A*. *muciniphila* is a mucin-degrading bacterium (Derrien et al., 2008), and colonic mucus thickness has been shown to be reduced with age (Elderman et al., 2017). An association between reduced small intestinal mucin-producing goblet cells in aged mice and the abundance of *A*. *muciniphila* has also been reported (Sovran et al., 2019). TLR ligands, including flagellin, have been reported to enhance colonic mucus thickness (Birchenough et al., 2016). Restoration of crypts in aged mice could restore goblet cell differentiation, creating a niche for *A*. *muciniphila*. Thus, changes to the crypts may be the cause of both the increased abundance of *A*. *muciniphila* and increased M cell density. Little is known of the unclassified *Turicibacter* species; however, *Turicibacteriaceae* are decreased in mice that lack the pro-inflammatory cytokine TNF-α (Jones-Hall et al., 2015) and increased in humans with rheumatoid arthritis (Chen et al., 2016). Increased TNF-α production may underlie macrophage dysfunction and microbiota dysbiosis in the aging intestine (Thevaranjan et al., 2017). Thus, the decreased *Turicibacter* observed in this study may be indicative of decreased aging-associated intestinal inflammation after exposure to a young microbiota. Whether reduced age-related inflammation promotes increased M cell maturation or is a consequence of increased M cell maturation is not known. An increased M cell density may also reduce the abundance of certain bacteria as was recently demonstrated for segmented filamentous bacteria (Lai et al., 2020). Interestingly, aged mice fed resistant starch, as a means to increase short chain fatty acid (SCFA) production, also show reduced *Turicibacter* and increased *A*. *muciniphila* abundance (Tachon et al., 2013), suggesting the changes observed in aged mice may not have been due to direct acquisition from the donor microbiota.

If the increase in *A*. *muciniphila* is independent of the effect on M cell maturation, it raises the question of to what extent the donor microbiota needs to come from young mice. Previous studies showing increased M cells after moving from SPF to conventional housing (Smith et al., 1987) may have been due to the immune system being challenged rather than the introduction of specific new species that induce M cells. Transfer of an aged microbiota into young mice has been shown to induce changes to Tfh cells in the Peyer's patches similar to those seen in aged mice given a young microbiota (Stebegg et al., 2019), suggesting that some of the observed changes to aged mice given young bedding may have been due to challenge of the immune system rather than a shift toward a young microbiota. However, it is important to note that transfer of an aged microbiota, not a young microbiota into germ-free mice, results in inflammation (Fransen et al., 2017). The much greater levels of variation between individual aged mice suggests that co-housed aged mice may be continually challenged by each other's microbiota under normal housing conditions. Housing aged mice on used bedding from a separate cohort of aged mice would help resolve this issue.

Beyond the effect on OLFM4 expressing cells in the crypts, altering the microbiota may have had additional effects on M cell maturation. M cell differentiation depends on RANK-RANKL signaling, and exogenous treatment of mice with RANKL can enhance the M cell density (Knoop et al., 2009) and increase susceptibility to M cell targeting pathogens (Donaldson et al., 2016). The density of M cells is also increased in mice that lack OPG, a decoy receptor for RANKL (Kimura et al., 2020). The RANK/RANKL/OPG axis is also important in osteoclast differentiation, which is required for bone development (Lacey et al., 1998). An association between the microbiota and bone health exists (Jones et al., 2018), and intestinal inflammation can affect systemic RANKL and OPG levels (Moschen et al., 2005) that may also have effects on M cell maturation in the Peyer's patches.

Although previous studies have suggested that systemic flagellin treatment stimulates CCL20 expression throughout the intestinal epithelium (Sirard et al., 2009), CCL20 was not significantly increased in the FAE in our study. Consistent with this, the ability of flagellin to induce CCL20 in enteroid cultures was modest in comparison with that induced by RANKL. In the intestinal epithelium TLR5 is predominantly expressed by Paneth cells (Price et al., 2018), which help maintain intestinal crypt stem cells. We have shown that macrophage depletion results in an altered Paneth cell phenotype that impairs M cell development (Sehgal et al., 2018). Paneth cell function is also defective in the aging intestine (Pentinmikko et al., 2019). The increase in OLFM4+ cells observed in the intestinal crypts of aged mice treated with flagellin may be due to direct stimulation of TLR5-expressing Paneth cells. However, other mechanisms, such as stimulation of hematopoietic cells (Kinnebrew et al., 2010), may also synergize with this to promote the large increase in M cell maturation observed after flagellin treatment. Further studies using Paneth cell or hematopoietic cell-specific deletions of TLR5 expression would confirm the relative importance of each cell type. Other bacterial ligands such as peptidoglycan, which signals through TLR2, have also been shown increase M cell uptake of microparticles (Chabot et al., 2006), suggesting the ability to restore M cell maturation in aged mice may not be limited to flagellin. Additionally, a number of other bacterial ligands are known to stimulate Paneth cells (Rumio et al., 2012, Vaishnava et al., 2008); however the effect of these on aged mice is yet to be tested.

How flagellin stimulation increases OLFM4 expression by stem cells in aged mice is unknown but may involve improved Wnt protein signaling, as exogenous Wnt signaling restores stem cell function in aged enteroids (Nalpareddy et al., 2017). Similar effects are observed after notum inhibition, a Wnt inhibitor upregulated in aged Paneth cells (Pentinmikko et al., 2019). Interestingly, flagellin stimulation increases expression of genes associated with mTORC1 signaling in enteroids (Price et al., 2018). Aging Paneth cells also have higher expression of genes associated with mTORC1 signaling, and inhibition of this pathway restores intestinal crypt cell function (Pentinmikko et al., 2019). Following flagellin stimulation, pS6, a downstream effector of mTORC1, was reduced in the intestinal crypts, suggesting that mTORC1 activity was reduced by flagellin stimulation. Although seemingly contradictory, this suggests that flagellin activation of mTORC1 may initiate autoregulatory pathways that inhibit mTORC1 activity (Yang et al., 2019). This may underlie the effect of flagellin on Paneth cells and the subsequent effects on M cell maturation and intestinal immunity.

In conclusion, our data suggest that the age-related decline in intestinal immunity can be reversed by boosting M cell numbers through manipulation of the microbiota or flagellin stimulation. Restoring the M-cell to IgA axis in the elderly could offset the harmful effects associated with the age-related changes to the microbiota and thus improve health. Additionally, this could be used to enhance responses to oral vaccination or improve the outcome of intestinal pathogen infections to which aged individuals are more susceptible. The observation that this may rely on improving intestinal crypt stem cell function means that treatments aimed at restoring the regenerative capacity of the aged intestine by modulating intestinal stem cells may have the added benefit of improving intestinal immunity.

### Limitations of the Study

One of the limitations of this study is that the aged mice were only housed on bedding from young mice, and thus the effect of confounding factors such as urine and stress cannot be excluded. To what extent the effects were dependent on a young microbiota rather than novel microbial stimulation also requires further study. Additionally, commensal intestinal microbiotas can vary between institutions and suppliers such that differential effects may be observed in mice housed in different institutes or purchased from different suppliers. No direct effect on M cell maturation was demonstrated for the particular bacterial families identified as correlating with M cell maturation. Although an increased number of OLFM4 expressing cells were observed in both mice given a young microbiota and flagellin treatment, evidence is now required to determine whether this is directly involved in increasing M cell maturation.

### Resource Availability

#### Lead Contact

Neil A. Mabbott; neil.mabbott@roslin.ed.ac.uk.

#### Materials Availability

Materials and protocols used in this study are available from the authors upon request.

#### Data and Code Availability

The bacterial 16S rRNA gene metabarcoding sequence files generated with the primers removed are publicly available through the European Nucleotide Archive (ENA) under the project accession number PRJEB36358 via the following URL: https://www.ebi.ac.uk/ena/data/view/PRJEB36358.

## Methods

All methods can be found in the accompanying Transparent Methods supplemental file.

## Supplemental Information

Document S1. Transparent Methods
