# Supplementary material for: Microbial Stimulation Reverses the Age-Related Decline in M Cells in Aged Mice
Source: iScience. 2020 May 11;23(6):101147. doi: 10.1016/j.isci.2020.101147 (PMC7251786; doi:10.1016/j.isci.2020.101147)
Supplement: Document S1. Transparent Methods [file mmc1.pdf]

iScience, Volume *23*

Supplemental Information

Microbial Stimulation Reverses the Age-Related Decline in M Cells in Aged Mice

David S. Donaldson, Jolinda Pollock, Prerna Vohra, Mark P. Stevens, and Neil A. Mabbott

Supplemental Information

TRANSPARENT METHODS

Mice

Male C57BL/6J mice were purchased from Charles River (Margate, UK). RANK$^{\Delta IEC}$ and RANK$^{F/F}$ mice (Rios et al., 2016) were bred at the University of Edinburgh. Mice were maintained in-house under specific pathogen-free conditions to the ages required. Young mice were used at 6-8 weeks old, aged mice were used at approx. 20-26 months old. All the experiments described in this study were first approved by The Roslin Institute's Ethical Review Committee, and were conducted under the authority of a UK Home Office project licence in full compliance with the Animals (Scientific Procedures) Act 1986.

Passive microbiota transfer

To facilitate the passive transfer of the faecal microbiota from young mice to aged mice, we housed aged mice for a 6 week period in cages containing used bedding that had previously been used to house young mice. To provide the donor bedding, young mice were removed from their caging and placed into a fresh cage with clean bedding. The aged mice were then housed in the empty used cage that previously been used to house the young mice. This was repeated twice weekly (every 3-4 d) for a 6 week period. Two cages of donor young mice were used and the aged mice were alternated between them. Groups of aged mice were housed on clean bedding that had not previously been used to house other mice as a control.

Bacterial 16S rRNA Gene Metabarcoding

Faecal samples were collected from aged mice before and at 4 and 6 wk after passive microbiota transfer and from young donor mice. DNA was extracted using a DNeasy PowerSoil Kit (Qiagen, Manchester, UK) as per the manufacturer's instructions and prepared for 16S rRNA gene sequencing, targeting the V3 hypervariable region, as described previously (Pollock et al., 2018). One library pool was constructed using equimolar concentrations of DNA from each of the included samples (n=39), as calculated using a fluorometric assay (Qubit dsDNA HS Assay kit, Thermo Fisher Scientific, Paisley, UK). Additionally, a mock bacterial community (20 Strain Even Mix Genomic Material ATCC®MSA-1002, ATCC, USA), a reagent-only control sample and two sham samples (empty tube controls exposed only to air in the room at the time of sampling) were included in the pool to assess sequencing error rate and background DNA contamination. Using the mock bacterial community data suggested the sequencing error rate was ~0.01%.

The library pool was quantified using the Quant-iT™ PicoGreen® double-stranded DNA Assay Kit (Thermo Fisher Scientific) to ensure adequate DNA yield for sequencing using the Illumina MiSeq (Illumina, Cambridge, UK) using V2 chemistry and generating 250 bp paired-end reads (Edinburgh Genomics, Edinburgh, UK). The primer sequences were first removed from the forward and reverse reads using cutadapt (Martin, 2011). The sequence files generated with the primers removed are publicly available through the European Nucleotide Archive (ENA) under the project accession number PRJEB36358.

Mothur (version 1.40.5) (Schloss et al., 2009) was then used to generate contiguous sequences, and to carry out sequence quality control and analysis as described by the software developers (URL: https://www.mothur.org/wiki/MiSeq_SOP; accessed February 2019). Unique sequences were binned using a database-

independent approach. A mean of 140,836 sequences were obtained per sample after quality control, with one sample being removed from the analysis due to a low number of sequences being retained. Files were subsampled to the lowest number of sequences obtained (n = 4101) for analysis. The Shannon Index was calculated for each sample to assess alpha diversity. To assess beta diversity, a distance matrix was constructed using Yue and Clayton theta similarity coefficients (Yue and Clayton, 2005). To visualise community similarities between groups, Non-Metric Multidimensional Scaling (NMDS) plots were compiled. The statistical significance of differences in clustering by treatments was assessed by analysis of molecular variance (AMOVA) (Excoffier et al., 1992). The statistical significance of variation between populations was tested using homogeneity of molecular variance (HOMOVA) (Stewart Jr. and Excoffier, 1996).

Systemic bacterial flagellin treatment

Mice were given 10 µg Ultrapure flagellin from *S.* Typhimurium (Invivogen, Toulouse, France) in sterile PBS by intra-peritoneal injection daily for 3 d.

***In vivo* Uptake of Fluorescent Nanobeads**

Mice were given a single oral gavage of 2 × 10^{11} of Fluoresbrite Yellow Green labelled 200 nm microbeads (Polysciences, Hirschberg an der Bergstrasse, Germany) in 200 µl PBS. Mice were culled 24 h later and Peyer's patches were snap-frozen in liquid nitrogen. Serial frozen sections (6 µm in thickness) were cut on a cryostat and counterstained with DAPI (4′,6-Diamidine-2′-phenylindole; Thermo Fisher Scientific).

The number of beads in the SED from 3-4 sections of two Peyer's Patches per mouse (*n*=3–4 mice/group; total 9–33 SED/mouse studied) were counted. Images of Peyer's patches SED regions were acquired using Nikon Eclipse E400 fluorescent microscope using Micro Manager (http://www.micro-manager.org). Tissue auto-fluorescence was subtracted from displayed images using ImageJ.

Bacterial Strains and Culture Conditions

An *aroA* deletion mutant of *Salmonella enterica* serovar Typhimurium 4/74 (ST4/74 Δ*aroA*; (Buckley et al., 2010) and *E. coli* K-12 strain DH5α were routinely cultured at 37°C in Luria-Bertani (LB) broth and on LB agar. ST4/74 Δ*aroA* was cultured with 50 μg ml^{-1} kanamycin. To aid visualization of infected mouse cells *in vivo*, both strains were electroporated with the plasmid pFPV25.1, which carries *gfpmut3A* under the control of the *rpsM* promoter resulting in the constitutive synthesis of GFP (Valdivia and Falkow, 1996). Following electroporation, strains were routinely cultured at 37°C in media supplemented with 100 μg ml^{-1} ampicillin to maintain pFPV25.1. The plasmid is known to be stable in *Salmonella* in bovine ileal loops *in vivo* over 12 h (Vohra et al., 2019).

Bacterial uptake into Ligated Peyer's Patches

For inoculation of murine ligated ileal loops, overnight cultures of GFP-expressing ST4/74 Δ*aroA* and *E. coli* K-12 were diluted to obtain approximately 10^9 colony forming units (CFU)/ml. Viable counts were confirmed retrospectively by plating of 10-fold serial dilutions of the cultures on LB agar containing 100 μg/ml ampicillin. Mice were anesthetised and a gut loop prepared centred on an individual Peyer's patch. Each

loop was inoculated with 100 µl of culture (~ 10^8 CFU). Mice were culled 1.5 h later and Peyer's patches from the loops snap-frozen in liquid nitrogen. Serial frozen sections (6 µm thickness) were cut on a cryostat and counterstained with DAPI. The number of GFP-expressing *E. coli* K-12 in the SED were counted directly in 6 sections of Peyer's patch per mouse (n=3–4 mice/group; total 9–16 SED/mouse studied). GFP-expressing ST4/74 Δ*aroA* was visualised by immunostaining with rabbit polyclonal anti-GFP and Alexa Flour 594 labelled anti-rabbit IgG (both Thermo Fisher Scientific). GFP-expressing ST4/74 Δ*aroA* in the SED were then counted in 12 sections of Peyer's patch per mouse (n=3–4 mice/group; total 9–56 SED/mouse studied). Images of Peyer's patches SED regions were acquired using Nikon Eclipse E400 fluorescent microscope using Micro Manager. Tissue auto-fluorescence was subtracted from displayed images using ImageJ software (https://imagej.nih.gov/ij/).

Bacteriological analysis of tissues

Mesenteric lymph nodes (MLNs) from infected mice were snap-frozen in liquid nitrogen. Tissues were thawed and then gently washed in PBS to remove non-adherent bacteria and weighed. A 10% homogenate was prepared in PBS using a Tissue Lyser II (Qiagen) and stainless steel beads. Ten-fold serial dilutions were plated both on LB agar and MacConkey agar containing 100 µg/ml ampicillin with or without 50 µg/ml kanamycin to differentiate between ST4/74 ΔaroA and *E. coli* K-12.

Oral Immunization with Horse Spleen Ferritin

Antigen-specific faecal IgA responses to an orally administered antigen were assessed in aged mice as previously described (Rios et al., 2016). Four weeks after the passive microbiota transfer was initiated, horse spleen ferritin (1mg/ml; Sigma,

Gillingham, UK) was orally administered to aged mice via drinking water on days 0–2 and 7–9. Faecal samples were collected 2 wk later and a 10% homogenate (w/v) prepared in PBS. Horse spleen ferritin-specific IgA levels in the supernatant of the faecal homogenates was determined by enzyme-linked immunosorbent assay in plates coated with horse spleen ferritin followed by detection with horseradish peroxidase-conjugated goat anti-mouse IgA (Southern Biotech, Birmingham, AL, USA) using BD TMB substrate reagent set (BD Biosciences, Oxford, UK) as the substrate. O.D. values were corrected for background using matched faecal samples collected immediately prior to the commencement of oral immunisation.

IHC Analysis

To detect M cells by whole-mount immunostaining Peyer's patches were first fixed using BD Cytofix/Cytoperm (BD Biosciences), and then immunostained with rat anti-mouse GP2 mAb (MBL International, Woburn, MA). Peyer's patches were then stained with Alexa Fluor 488-conjugated anti-rat IgG Ab and Alexa Fluor 647-conjugated phalloidin to detect F-actin (both Thermo Fisher Scientific).

Peyer's patches and small intestines were also snap-frozen at the temperature of liquid nitrogen, and 6 μm serial frozen sections cut using a cryostat. To detect MNP, sections were immunostained with hamster anti-mouse CD11c mAb (clone N418, Thermo Fisher Scientific) and rat anti-mouse CD68 mAb (clone FA-11, Biolegend, London, UK). To detect Spi-B, paraformaldehyde-fixed frozen sections were treated with citrate buffer (pH 7.0, 121°C, 5 min) before immunostaining with sheep anti-mouse Spi-B polyclonal Ab (R&D Systems, Abingdon, UK). CCL20, OLFM4 and pS6 were detected in paraformaldehyde-fixed frozen sections using goat anti-mouse CCL20 polyclonal Ab (R&D Systems), rabbit anti-mouse OLFM4 mAb (clone D6Y5A)

or rabbit anti-phospho-S6 ribosomal protein mAb (clone D68F8) (both Cell Signalling Technology, London, UK). Sections were subsequently immunostained with species-specific secondary antibodies coupled to Alexa Fluor 488 (green) or Alexa Fluor 594 (red) dyes (Thermo Fisher Scientific). Cell nuclei were detected using DAPI. Sections were mounted in fluorescent mounting medium (DAKO, Stockport, UK) prior to imaging on a Zeiss LSM710 confocal microscope (Zeiss, Cambourne, UK).

Image Analysis

Digital microscopy images were analysed using ImageJ software as described previously (Inman et al., 2005). Background intensity thresholds were first applied using an ImageJ macro which measures pixel intensity across all immunostained and non-stained areas of the images. The obtained pixel intensity threshold value was then applied in all subsequent analyses. Next, the number of pixels of each color (black, red, green, yellow etc.) were automatically counted and presented as a proportion of the total number of pixels in each area under analysis. To analyse immunostaining in FAE and SED regions, 3-8 images were routinely analysed/mouse. For wholemounts, GP2+ cells were counted in 4-7 FAE/mouse. Cell counting in sections of FAE (Spi-B+ cells, CD11c+ cells) was performed on 1-15 images/mouse. The number of OLFM4+ cells was counted in 28-74 crypts/mouse.

***In Vitro* Enteroid Cultivation**

Intestinal crypts were dissociated from mouse small intestine using Gentle Cell Dissociation Reagent (Stemcell Technologies, Cambridge, UK). The crypts were then re-suspended in Intesticult medium (Stemcell Technologies) at $4x10^3$ crypts/ml and mixed 1:1 with Growth Factor Reduced Matrigel matrix (Corning, Flintshire, UK). Next,

50 μl Matrigel plugs were plated in pre-warmed 24-well plates and allowed to settle, before addition of 600 μl of pre-warmed Intesticult medium and subsequently cultured at 37°C in a 5% CO_2 atmosphere. Fresh medium was replaced every 2 d of cultivation and the enteroids passaged after 7 days of culture. Where indicated, enteroids prepared from the 1st passage were treated with either RANKL (50ng/ml, Biolegend), ultrapure flagellin from *S.* Typhimurium (100 ng/ml; Invivogen) or in combination. For each experimental condition, enteroids were cultivated in triplicate and repeated using enteroids from three independent animals.

Real-time Quantitative PCR (RT–qPCR) Analysis of mRNA Expression

For mRNA extraction, enteroids were incubated in Cell Recovery Solution (Corning) for 1 h at 4 °C. Total RNA was then isolated using RNeasy Mini Kit (Qiagen) followed by removal of genomic DNA and cDNA synthesis using SuperScript IV VILO Master Mix with ezDNase Enzyme (Thermo Fisher Scientific) both as per manufacturer's instructions. PCR was performed using the Platinum-SYBR Green qPCR SuperMix-UDG kit (Thermo Fisher Scientific) and the Stratagene Mx3000P real-time qPCR system (Stratagene, CA, USA). Primers used are listed in Table S1.

Table S1: Primers used in RT-qPCR analyses

Gene	*Forward*	*Reverse*
Ccl20	5'-CGACTGTTGCCTCTCGTACA-3'	5'-AGCCCTTTTCACCCAGTTCT-3'
Gapdh	5'-GGGTGTGAACCACGAGAAAT-3'	5'-CCTTCCACAATGCCAAAGTT-3'
Gp2	5'-GATACTGCACAGACCCCTCCA-3'	5'-GCAGTTCCGGTCATTGAGGTA-3'
Olfm4	5'-TGGCCCTTGGAAGCTGTAGT-3'	5'-ACCTCCTTGGCCATAGCGAA-3'
Sox8	5'-TCCGTTGCTCTCCGGTTT-3'	5'-GCCCATTCTCTCCTTTGTCCT-3'
Spib	5'-AGCGCATGACGTATCAGAAGC-3'	5'-GGAATCCTATACACGGCACAGG-3'

Statistical Analyses

Details of all group/sample sizes and experimental repeats are provided in the figure legends. Statistical analyses were performed in Prism 6 (Graphpad Software, San Diego, CA). Details of tests used are provided in the figure legends. In instances where there was evidence of non-normality (identified by the D'Agostino & Pearson omnibus, Shapiro-Wilk or Kolmogorov–Smirnov normality test), data were analysed using appropriate non-parametric tests. Values of $P < 0.05$ were accepted as significant.

KEY RESOURCES TABLE

REAGENT or RESOURCE	SOURCE	IDENTIFIER
**Antibodies**		
Rabbit polyclonal anti-GFP	Thermo Fisher Scientific	Cat #A-11122; RRID:AB_22156
Rat anti-mouse GP2 (2F11-C3)	MBL International	Cat #D278-3; RRID:AB_10598188
Alexa Flour 488-conjugated hamster anti-mouse CD11c mAb (N418)	Thermo Fisher Scientific	Cat #53-0114-82; RRID:AB_469903
Rat anti-mouse CD68 mAb (FA-11)	Biolegend	Cat #137001; RRID:AB_2044003
Sheep anti-mouse Spi-B polyclonal Ab	R&D Systems	Cat #AF7204; RRID:AB_10995033
Goat anti-mouse CCL20 polyclonal Ab	R&D Systems	Cat #AF760; RRID:AB_355580
Rabbit anti-mouse OLFM4 mAb (D6Y5A)	Cell Signaling Technology	Cat #39141S; RRID:AB_2650511
Rabbit anti-phospho-S6 ribosomal protein (D68F8)	Cell Signaling Technology	Cat #5364S; RRID:AB_10694233
Alexa Fluor 488-conjugated Goat anti-rat IgG	Thermo Fisher Scientific	Cat #A-11006; RRID:AB_141373
Alexa Fluor 647-conjugated Goat anti-rat IgG	Thermo Fisher Scientific	Cat #A-21247; RRID:AB_141778
Alexa Fluor 488-conjugated Donkey anti-sheep IgG	Thermo Fisher Scientific	Cat #A-11015; RRID:AB_141362
Alexa Fluor 594-conjugated Rabbit anti-goat IgG	Thermo Fisher Scientific	Cat #A-11080; RRID:AB_2534124
Alexa Flour 594-conjugated Goat anti-rabbit IgG	Thermo Fisher Scientific	Cat #A-11012; RRID:AB_141359
Horseradish peroxidase-conjugated goat anti-mouse IgA	Southern Biotech	Cat #1040-05; RRID:AB_2714213
**Bacterial and Virus Strains**		

aroA mutant of *Salmonella enterica* serovar Typhimurium strain 4/74 with spontaneous nalidixic acid resistance and an *aph* (kanamycin resistance gene) inserted at the site of *aroA* deletion.	Buckley et al., 2010	ST4/74 Δ*aroA*
E. coli K-12 strain DH5α	Invitrogen	Cat #18265017
**Chemicals, Peptides, and Recombinant Proteins**		
Ultrapure flagellin from *S.* Typhimurium	Invivogen	Cat #tlrl-epstfla
Fluoresbrite Yellow Green labelled 200 nm microbeads	Polysciences	Cat #17151-10
Horse spleen ferritin	Sigma	Cat #F4503
BD Cytofix/Cytoperm Fixation and Permeabilization Solution	BD Biosciences	Cat #554722
DAPI (4′,6-Diamidine-2′-phenylindole)	Thermo Fisher Scientific	Cat #D1306
Alexa Fluor 647-conjugated phalloidin	Thermo Fisher Scientific	Cat #A22287
Fluorescent mounting medium	DAKO	Cat #S302380-2
BD TMB substrate reagent set	BD Biosciences	Cat #13459936
Gentle Cell Dissociation Reagent	Stemcell Technologies	Cat #07174
IntestiCult™ Organoid Growth Medium (Mouse)	Stemcell Technologies	Cat #06005
Matrigel Matrix, Growth Factor Reduced (GFR), Phenol Red-Free	Corning	Cat #356231
RANKL	Biolegend	Cat #577102
Cell Recovery Solution	Corning	Cat #354253
**Critical Commercial Assays**		
DNeasy PowerSoil Kit	Qiagen	Cat #12888-100
Qubit dsDNA HS Assay kit	Thermo Fisher Scientific	Cat #Q32854
Quant-iT PicoGreen double-stranded DNA Assay Kit	Thermo Fisher Scientific	Cat #P11496
RNeasy Mini Kit	Qiagen	Cat #74104
SuperScript IV VILO Master Mix with ezDNase Enzyme	Thermo Fisher Scientific	Cat #11766050
Platinum-SYBR Green qPCR SuperMix-UDG kit	Thermo Fisher Scientific	Cat #11733038
**Deposited Data**		
Bacterial 16S rRNA Gene Sequencing	This paper	ENA: PRJEB36358
**Experimental Models: Organisms/Strains**		
C57BL/6J	Charles River	Strain #027
B6.Cg-*Tnfrsf11a*$^{tm1.1Irw}$/J (RANK$^{F/F}$)	The Jackson Laboratory	JAX: 027495
B6.Cg-Tg(Vil1-cre)997Gum/J (Villin-cre)	The Jackson Laboratory	JAX: 004586
**Oligonucleotides**		

See Table S1		
**Recombinant DNA**		
pFPV25.1	Valdivia and Falkow, 1996	Addgene: 20668
20 Strain Even Mix Genomic Material	ATCC	ATCC: MSA-1002
**Software and Algorithms**		
Mothur (version 1.40.5)	Schloss et al., 2009	https://mothur.org
Micro Manager	N/A	http://www.micro-manager.org
ImageJ software	N/A	https://imagej.nih.gov/ij/
Prism 6	Graphpad Software	https://www.graphpad.com/scientific-software/prism/

References

Buckley, A.M., Wang, J., Hudson, D.L., Grant, A.J., Jones, M.A., Maskell, D.J., and Stevens, M.P. (2010). Evaluation of live-attenuated *Salmonella* vaccines expressing *Campylobacter* antigens for control of *C. jejuni* in poultry. Vaccine *28*, 1094-1105.

Excoffier, L., Smouse, P.E., and Quattro, J.M. (1992). Analysis of molecular variance inferred from metric distances among DNA haplotypes: application to human mitochondrial DNA restriction data. Genetics *131*, 479-491.

Inman, C.F., Rees, L.E.N., Barker, E., Haverson, K., Stokes, C.R., and Bailey, M. (2005). Validation of computer-assisted, pixel-based analysis of multiple-colour immunofluorescence histology. J Immunol Met *302*, 156-167.

Martin, M. (2011). Cutadapt removes adapter sequences from high-throughput sequencing reads. EMBnetjournal *17*, 10-12.

Pollock, J., Gally, D.L., Glendinning, L., Tiwari, R., Hutchings, M.R., and Houdjik, J.G.M. (2018). Analysis of temporal fecal microbiota dynamics in weaner pigs with and without exposure to enterotoxigenic *Escherichia coli*. Journal of Animal Science *96*, 3777-3790.

Rios, D., Wood, M.B., Li, J., Chassaing, B., Gewirtz, A.T., and Williams, I.R. (2016). Antigen sampling by intestinal M cells is the principal pathway initiating mucosal IgA production to commensal enteric bacteria. Mucosal Immunol *9*, 907-916.

Schloss, P.D., Westcott, S.L., Ryabin, T., Hall, J.R., Martmann, M., Hollister, E.B., Lesniewski, R.A., Oakley, B.B., Parks, D.H., Robinson, C.J.*, et al.* (2009). Introducing mothur: open-source, platform-independent, community-supported software for describing and comparing microbial communities. Appl Environ Microbiol *75*, 7537-7541.

Stewart Jr., C.N., and Excoffier, L. (1996). Assessing population genetic structure and variability with RAPD data: applicaton to *Vaccinium macrocarpon* (American Cranberry). J Evol Biol *9*, 153-171.

Valdivia, R.H., and Falkow, S. (1996). Bacterial genetics by flow cytometry: rapid isolation of *Salmonella typhimurium* acid-inducible promoters by differential fluorescence induction. Mol Microbiol *22*, 367-378.

Vohra, P., Vrettou, C., Hope, J.C., Hopkins, J., and Stevens, M.P. (2019). Nature and consequences of interactions between *Salmonella enterica* serovar Dublin and host cells in cattle. Vet Res *50*, 99.

Yue, J.C., and Clayton, M.K. (2005). A similarity measure based on species proportions. Commun Stats Theory Met *34*, 2123-2131.
